# Predictive Mapping of Topsoil Organic Carbon in an Alpine Environment Aided by Landsat TM

**DOI:** 10.1371/journal.pone.0139042

**Published:** 2015-10-16

**Authors:** Renmin Yang, David G. Rossiter, Feng Liu, Yuanyuan Lu, Fan Yang, Fei Yang, Yuguo Zhao, Decheng Li, Ganlin Zhang

**Affiliations:** 1 State Key Laboratory of Soil and Sustainable Agriculture, Institute of Soil Science, Chinese Academy of Sciences, Nanjing 210008, China; 2 University of the Chinese Academy of Sciences, Beijing 100049, China; 3 Department of Crop & Soil Sciences, Cornell University, Ithaca, NY 14853, United States of America; Beijing Normal University, CHINA

## Abstract

The objective of this study was to examine the reflectance of Landsat TM imagery for mapping soil organic Carbon (SOC) content in an Alpine environment. The studied area (ca. 3*10^4^ km^2^) is the upper reaches of the Heihe River at the northeast edge of the Tibetan plateau, China. A set (105) of topsoil samples were analyzed for SOC. Boosted regression tree (BRT) models using Landsat TM imagery were built to predict SOC content, alone or with topography and climate covariates (temperature and precipitation). The best model, combining all covariates, was only marginally better than using only imagery. Imagery alone was sufficient to build a reasonable model; this was a bit better than only using topography and climate covariates. The Lin’s concordance correlation coefficient values of the imagery only model and the full model are very close, larger than the topography and climate variables based model. In the full model, SOC was mainly explained by Landsat TM imagery (65% relative importance), followed by climate variables (20%) and topography (15% of relative importance). The good results from imagery are likely due to (1) the strong dependence of SOC on native vegetation intensity in this Alpine environment; (2) the strong correlation in this environment between imagery and environmental covariables, especially elevation (corresponding to temperature), precipitation, and slope aspect. We conclude that multispectral satellite data from Landsat TM images may be used to predict topsoil SOC with reasonable accuracy in Alpine regions, and perhaps other regions covered with natural vegetation, and that adding topography and climate covariables to the satellite data can improve the predictive accuracy.

## Introduction

As a key component of carbon fluxes between terrestrial ecosystems and the atmosphere, soil carbon has received considerable attention in a growing number of studies motivated in part by the Kyoto protocol for controlling the concentrations of greenhouse gasses [1]. Because of its influence on soil fertility, soil structure, soil biological processes and soil hydraulic properties, soil organic carbon (SOC) is a crucial soil property for soil management and a priority for research.

It is effectively impossible to sample and analyze enough points to map SOC over large areas, especially in difficult topography such as Alpine environments. Digital soil mapping (DSM) methods provide a rapid and inexpensive way to estimate SOC content over large areas from limited samples and environmental covariates. Most DSM methods are based on soil-landscape models [2–5], which build quantitative relationships between SOC and easily-obtained environmental covariates, including topography, climate, parent material and organisms.

A major DSM covariate related to SOC, especially in natural areas, is vegetation intensity. This can be estimated by remote sensing-derived products such as vegetation maps, land use maps, biomass maps and vegetation indexes, and has been widely used in SOC prediction by DSM methods [6–9]. Some attempts have been made to map SOC content from satellite multispectral imagery, including 4-m IKONOS [10], 10 and 20 m SPOT [11] and 15 and 30 m Landsat TM [12–16]. These studies generally estimated SOC content from reflectance values of image bands using equations derived by linear regression in areas with homogeneous soil types or cultivated agricultural fields, and on bare soil surfaces or partial vegetation covered areas to minimize the vegetation influence. However, it might be possible to directly use vegetation reflectance to predict SOC content, because SOC variability is influenced by vegetation, especially topsoil SOC in natural environments [17], and has been shown to be well-correlated with above-ground biomass [18].

Most DSM exercises in high-relief areas use topography parameters derived from digital elevation models (DEM) as the primary covariates [5]. And indeed topography position may have a substantial effect on SOC. However, imagery provides a direct representation of the surface, and if it can be shown to be closely-related to SOC, DSM for this property could be considerably simplified.

Linear regression models, as used in previous studies, have several limitations. The most obvious deficiency is that they cannot model nonlinear relationships between soil properties and predictors. By contrast, regression trees [19] break down the model into a tree in which each node is labeled using response value and split by predictive variables. These however only have one solution and are not robust to small changes in data [20]. An attractive alternative is boosted regression tree models (BRT). These combine many simple trees to improve the predictive performance and especially to ensure robustness [20–22]. BRT can deal with various data types, missing values, outliers, irrelevant predictors and interactions between predictors and provides information to evaluate, summarize and interpret the fitted model [23]. Owing to these advantageous properties, BRT have been used in various scientific fields such as environmental science [24], ecology [21, 25], remote sensing [26, 27], and soil science [4, 28–32].

No study has evaluated the predictive performance of multispectral satellite imagery in mapping SOC content by using DSM methods over a large, natural vegetation covered, Alpine area. The present study is of the Alpine environment at the northeast edge of the Tibetan Plateau, which is the largest high-altitude ecosystem. This area consists of Qilian Mountains. Complex mountain topography leads to a variety vegetation types in this region. Together with low temperature, there is a significant amount of SOC pool in the Tibetan Plateau due to low decomposition rates. Therefore, SOC in this area is thought to be especially sensitive to global climate change, grassland degradation and human activities but very critical to ecosystem functions [33].

The aim of this study is to evaluate the potential of using BRT and Landsat TM imagery for mapping topsoil (0–20 cm) organic carbon content in areas with natural Alpine vegetation cover. The specific objectives were: (1) relating Landsat TM reflectance to topsoil organic carbon content using BRT; (2) measuring the success of this method and its potential for wider application; (3) attempting to explain the results by physical principles of remote sensing. We evaluate success by comparing models using only Landsat TM imagery to models incorporating topography parameters, as well as full models with both imagery and topography.

## Materials and Methods

### Ethics Statement

No specific permissions were required for each sampling location in our study area. And no endangered or protected species were involved in the field studies.

### Site Description

**Fig 1:**
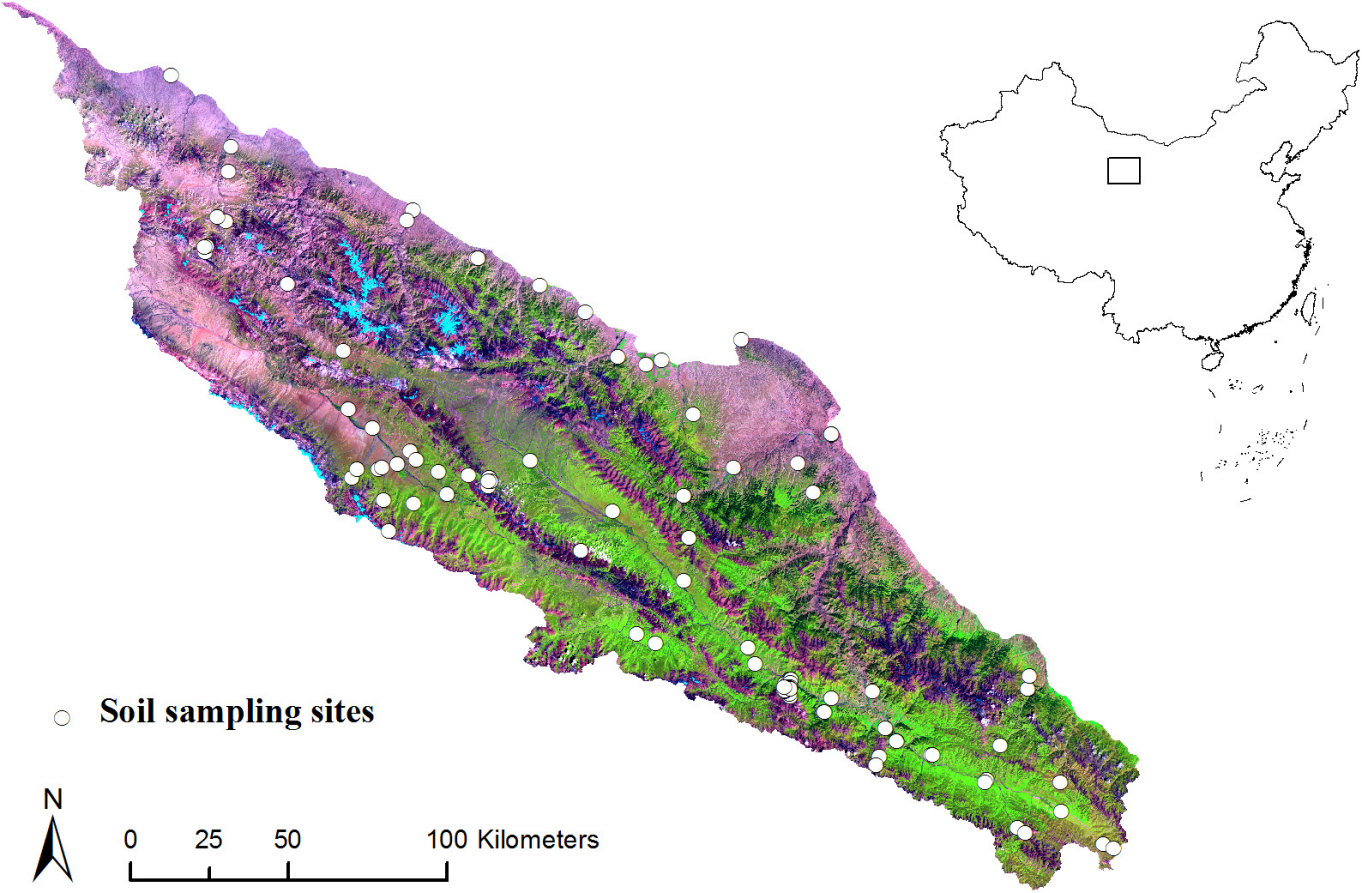
Location of study area and sample sites. Background is a Landsat TM color composite of the study area (red: short-wave infrared band 5; green: near infrared band 4; blue: visible band 3).

The study area is located in the margin of the Tibetan Plateau, northwestern China. It covers an area of approximately 3*10^4^ km^2^ between latitudes 37.71° and 40.03° N and longitudes 96.78° and 101.2° E (Fig 1). This region is dominated by the Qilian Mountains with high relief (1,684 to 4,600 m above sea level), and is the source of the Heihe River, the second largest inland river in China. This variation in topography is accompanied with variation in soil types, including Inceptisols, Entisols, and Histosols according to Soil Taxonomy [34]. Parent material is dominated by slope deposit, alluvial and moraine materials. The area is sparsely settled with no cities. Land use is mainly grazing lands, with some farmlands scattered near towns. The southeastern grasslands have high vegetation cover, in contrast to the northwestern and northern grasslands.

### Soil samples

A purposively sampling strategy was used to identify sample sites to represent the variability of elevation, climate, land use and parent material. We conducted soil survey and collected soil samples from one hundred and five (105) soil profiles in the summers of 2012 and 2013 (Fig 1). Locations were recorded using a handheld global positioning system (GPS). During field sampling, some of designed sites cannot be easily achieved due to the poor accessibility. These sampling locations were replaced with selected locations by expert judgment. Profiles were described by pedogenic horizons to a depth of 1.2 m or shallower if reached bedrock. About 1 kg soil was taken from each identified horizon. In the laboratory, all samples were air-dried and then sieved at 2 mm. The SOC content (g kg^-1^) of these samples was determined by Walkley-Black wet combustion method [35]. The SOC content of the top 20 cm was then computed by a depth-weighted average function (Table 1). The SOC content was log-transformed to minimize the right-skew of the untransformed variable for better modeling.

**Table 1:** Summary statistics of soil samples at 0–20 cm and environmental variables of the samples sites.

Property	Name	Unit	Min	Median	Mean	Max	SD	Skewness	Kurtosis
Soil	SOC	g kg^-1^	1.90	31.62	41.85	154.21	36.54	1.31	4.11
Topography	Elevation	m	1851	3342	3235	4357	589	-0.58	2.89
	Slope	degree	0.49	11.93	12.47	30.85	7.52	0.49	2.64
	Aspect	degree	8.53	60.40	70.82	165.45	45.24	0.42	2.01
	CA	m^2^ m^-1^	1.19	24.33	154.67	1590.23	294.39	3.12	13.84
	TWI		3.79	5.48	5.79	9.89	1.41	0.61	2.73
Climate	MAP	mm	105	302	289	454	75.69	-0.48	2.93
	MAT	degree celsius	-7.13	-1.19	-1.12	5.91	3.18	0.28	2.63
Landsat TM	B3	digital number	20.78	34.56	39.94	89.67	18.12	1.11	3.24
	B4	digital number	42.67	83.44	84.75	121.11	16.33	0.02	2.76
	B5	digital number	42.78	97.44	99.35	153	21.77	0.44	2.85
	NDVI		0	0.41	0.37	0.7	0.22	-0.38	1.71

Notes: SOC, Soil organic carbon; CA, catchment area; TWI, SAGA wetness index; MAP, mean annual precipitation; MAT, mean annual temperature; B3, Landsat TM band 3; B4, Landsat TM band 4; B5, Landsat TM band 5; NDVI, normalized difference vegetation index.

### Topography and climate variables

Covariables were selected as proxies for presumed soil-forming factors (Table 1). A digital elevation model (DEM) was acquired from Shuttle Radar Topography Mission DEM (SRTM 2009) with 90 m resolution. Elevation, slope and aspect were determined using spatial analysis tools in ArcGIS 10.0 (ESRI Inc., USA). Two second derivatives, catchment area (CA) and SAGA wetness index (TWI), were derived in the SAGA GIS software [36]. Aspect was expressed as absolute 0 to 180° to represent face from north to south. Climate data was obtained from meteorological stations. Mean annual temperature (MAT) and mean annual precipitation (MAP) over thirty years, were derived as a 1 km grid from six hundred and seventy-three meteorological stations in China.

### Landsat TM

Landsat 5 TM imagery was acquired from the Cold and Arid Regions Sciences Data Center, Lanzhou [37]. In order to cover the spatial domain of study area, 21 images had been acquired from July to September (growing season) in 2010 with cloud cover less than 10%. The images were relief-corrected by polynomial geometric precision correction method and then mosaicked and trimmed to cover the study area. Landsat TM visible red Band 3 (B3, 0.63–0.69μm), near infrared Band 4 (B4, 0.76–0.96μm) and short-wave infrared Band 5 (B5, 1.55–1.75μm) were retained to represent the “organisms” soil forming factor. These three TM bands are responsive to vegetation growth, coverage and biomass. Vegetation intensity was represented by the Normalized Difference Vegetation Index (NDVI), (B4-B3)/ (B4+B3).

### Prediction model

We build three models with different combinations of predictive variables using BRT. The first model (MA) included all predictors. The second model (MB) included only topography and climate variables, and the third model (MC) included only Landsat TM imagery (B3, B4, B5 and NDVI). This allows us to evaluate the relative importance of imagery in this DSM context.

In fitting a BRT model, four parameters have to be optimized: the bag fraction (BF), the learning rate (LR), the tree complexity (TC) and the number of trees (NT). BF specifies the proportion of data used in each model; the more data used, the less the stochastic, i.e., the more similar are the trees [20]. LR is also called the shrinkage parameter; it determines the influence of each single tree to the final model. TC controls whether interactions between variables are fitted. NT is determined by the combination of LR and TC and is not separately specified. At least 1000 trees were recommended in fitting BRT models [20]. Various combinations of these parameters were tested to determine the optimal settings for the best predictive performance using 10-fold cross-validation, resulting in the combination of LR, TC and BF as 0.0025, 9, and 0.75 respectively. The relative importance of the predictors can be assessed by averaging the number of times a variable selected for splitting and the squared improvement resulting from these splits [20, 23]. Data mining and modeling tasks were performed in R [38], using a BRT script provided by Elith et al. [20].

The performance of the BRT model was evaluated using 10-fold cross-validation. Four indices were used: the mean absolute prediction error (MAE) measuring the average prediction bias, the root mean square error (RMSE) measuring the overall quality of the prediction, the coefficient of determination (R^2^) measuring the strength of the linear relationship between the predicted and observed values, Lin’s concordance correlation coefficient (LCCC) measuring the degree of predicted and observed values follow the 45° line [39] and the relative improvement (RI) measuring improvement of one model over the other. These measurements are defined as:

$$\text{MAE}=\frac{1}{n}\sum_{i=1}^{n}\left|P_{i}-O_{i}\right|$$ (1)

$$\text{RMSE}=\sqrt{\frac{1}{n}\sum_{i=1}^{n}{\left(P_{i}-O_{i}\right)}^{2}}$$ (2)

$$R^{2}=\frac{\sum_{i=1}^{n}{\left(P_{i}-\bar{O}\right)}^{2}}{\sum_{i=1}^{n}{\left(O_{i}-\bar{O}\right)}^{2}}$$ (3)

$$\text{LCCC}=\frac{2\text{r}\sigma_{o}\sigma_{p}}{\sigma_{o}^{2}+\sigma_{p}^{2}+{\left(\bar{O}-\bar{P}\right)}^{2}}$$ (4)

$$\text{RI}=\frac{{\text{RMSE}}_{1}\;-\;{\text{RMSE}}_{2}}{{\text{RMSE}}_{1}}\times 100$$ (5)

Where *P*_*i*_ and *O*_*i*_ are the predicted and observed values for *i*th observation; $\bar{P}$ and $\bar{O}$ are the means for the predicted and observed values; $\sigma_{p}^{2}$ and $\sigma_{o}^{2}$ are the variances of predicted and observed values; *r* is the Pearson correlation coefficient between the predicted and observed values and 1 and 2 represent two models.

## Results and Discussion

### Soil organic carbon content and its relation with predictors

The average soil organic carbon content in the topmost 20 cm in our study is 41.85 g kg^-1^ (Tab. 1), which is close to the average value of 52.4 g kg^-1^ in Tibetan grasslands by Shi et al. [18]. Compared with previous studies in natural grasslands in the Inner Mongolia [40, 41], the SOC contents are higher in Tibetan grasslands. The SOC content in our study is slightly higher than that of 38.5 g kg^-1^ in Chinese grasslands soils reported by Xie et al. [42]. Alpine grasslands on the Tibetan Plateau are one of the most important ecosystems in China, containing about 23% China’s SOC storage and about 2.5% of the global soil carbon pool [43]. Yang et al. [44] reported that SOC in the upper 30 cm is about 68% of total SOC in the upper 1 m in the Tibetan grasslands.

Linear correlations between SOC and quantitative predictors are shown in Table 2. SOC was positively correlated with elevation (r = 0.50) and negatively correlated with aspect, expressed as northness (r = -0.22). SOC was positively correlated with MAP (r = 0.74) and negatively correlated with MAT (r = -0.38). Of more interest for our study, correlations with imagery were all significant. The relation with NDVI in this natural area is expected; the slightly higher correlation with the single red band (B3) is somewhat surprising. Predictors within each group (topography, climate, imagery) and between groups had some colinearity.

**Table 2:** Pearson correlation analysis between ln(SOC) and environmental variables based on 105 samples.

	ln(SOC)	Elevation	Slope	Aspect	CA	TWI	MAP	MAT	B3	B4	B5
Elevation	**0.50**										
Slope	0.14	0.03									
Aspect	**-0.22**	0.16	**-0.19**								
CA	-0.09	0.06	**-0.44**	0.10							
TWI	-0.17	-0.07	**-0.87**	0.16	**0.62**						
MAP	**0.74**	**0.71**	-0.02	0.11	-0.07	-0.01					
MAT	**-0.38**	**-0.98**	0.05	**-0.19**	-0.14	-0.01	**-0.60**				
B3	**-0.82**	**-0.44**	-0.09	0.11	0.11	0.12	**-0.75**	**0.34**			
B4	**0.29**	-0.10	**-0.25**	0.13	0.12	**0.21**	**0.23**	0.12	**-0.34**		
B5	**-0.59**	**-0.37**	**-0.23**	**0.26**	0.14	**0.23**	**-0.49**	**0.29**	**0.78**	0.06	
NDVI	**0.79**	**0.29**	-0.01	-0.08	-0.04	-0.02	**0.67**	**-0.19**	**-0.91**	**0.68**	**-0.56**

Notes: CA, catchment area; TWI, SAGA wetness index; MAP, mean annual precipitation; MAT, mean annual temperature; B3, Landsat TM band 3; B4 Landsat TM band 4; B5, Landsat TM band 5; and NDVI, normalized difference vegetation index.Significant relationship between two variables with p<0.05 shown in bold.

### SOC content prediction

Three BRT models were fitted to the top 20 cm SOC content (Table 3). The MA model (with variables of topography, climate and Landsat TM imagery) outperformed the MB model (with topography plus climate) and the MC model (with variables of Landsat TM), offering the highest value of R^2^ (0.73) and LCCC (0.85) and the lowest values of MAE (0.4) and RMSE (0.52). This was expected–in general, the more predictors, the better the model. The interesting result here is that the MC model, using only imagery, was almost as successful as the full model (R^2^ = 0.69, LCCC = 0.82, MAE = 0.42, RMSE = 0.56), and considerably better than the model with no imagery. The RI showed that predictive performance was improved considerably (RMSE 14.7% lower) by adding remote sensing imagery (MA vs. MB); these results are consistent with the significant correlations between imagery and SOC (Table 2). By contrast, the improvement was much less (RMSE only 7.1% lower) when adding topography and climate variables to the imagery-only model (MA vs. MC). This is because, in this Alpine environment, much of the variability in topography and climate is correlated with imagery (Table 2), i.e., the imagery varies with these. For example, high elevations have bare rocks, north-facing slopes have less vegetation, etc.

**Table 3:** Predictive quality of three boosted regression tree (BRT) models for ln(SOC).

Model	Index	Min	1st Quartile	Mean	Median	3rd Quartile	Max
MA	MAE	0.38	0.39	0.4	0.4	0.41	0.43
	RMSE	0.5	0.51	0.52	0.52	0.53	0.55
	R^2^	0.67	0.71	0.73	0.73	0.74	0.76
	LCCC	0.83	0.84	0.85	0.85	0.85	0.86
MB	MAE	0.44	0.46	0.48	0.48	0.48	0.49
	RMSE	0.58	0.59	0.61	0.61	0.61	0.62
	R^2^	0.57	0.62	0.64	0.64	0.64	0.65
	LCCC	0.75	0.75	0.76	0.76	0.79	0.8
MC	MAE	0.4	0.41	0.42	0.42	0.43	0.44
	RMSE	0.53	0.55	0.56	0.56	0.57	0.58
	R^2^	0.65	0.68	0.69	0.7	0.72	0.75
	LCCC	0.8	0.81	0.82	0.82	0.83	0.84

Notes: MA, Topography + climate + Landsat TM; MB, Topography + climate; MC, Landsat TM; MAE, the mean absolute error; RMSE, the root mean squared error; R^2^, the coefficient of determination; LCCC, Lin’s concordance correlation coefficient.

The good predictive relations due to Landsat TM imagery alone can be explained ecologically and by the relation of imagery to these ecologic factors. Shi et al. [18] studied patterns and controls of topsoil (0–20 cm) organic and inorganic C of grassland ecosystems in the Inner Mongolia and the Tibetan Plateau in China. They found that the spatial patterns of topsoil SOC were mainly controlled by biotic processes, reflected in the vegetation type. This is consistent with many studies that show that vegetation is the main source of SOC (e.g., Jobbágy & Jackson, [17]). Table 2 shows that elevation determines climate characteristics in this region, which was significantly correlated with MAP (r = 0.71) and MAT (r = -0.98).

Aspect was only slightly correlated with MAT. Aspect influences microclimate only [45]. Therefore, the variability in topography can be substantially explained by precipitation and temperature, but in this case MAP was well-correlated with imagery, specifically NDVI (r = 0.67); the correlation with MAT was not so close (r = 0.19). This is consistent with the results of Ma et al. [46] who found plants under cold and humid environments usually have high productivity in Chinese grasslands. Jobbágy & Jackson. [17] found that high precipitation indicates high productivity of vegetation. For SOC, high productivity of grassland means more organic materials input in soils. Low temperature causes slow decomposition rates of SOC [17, 47].

**Fig 2:**
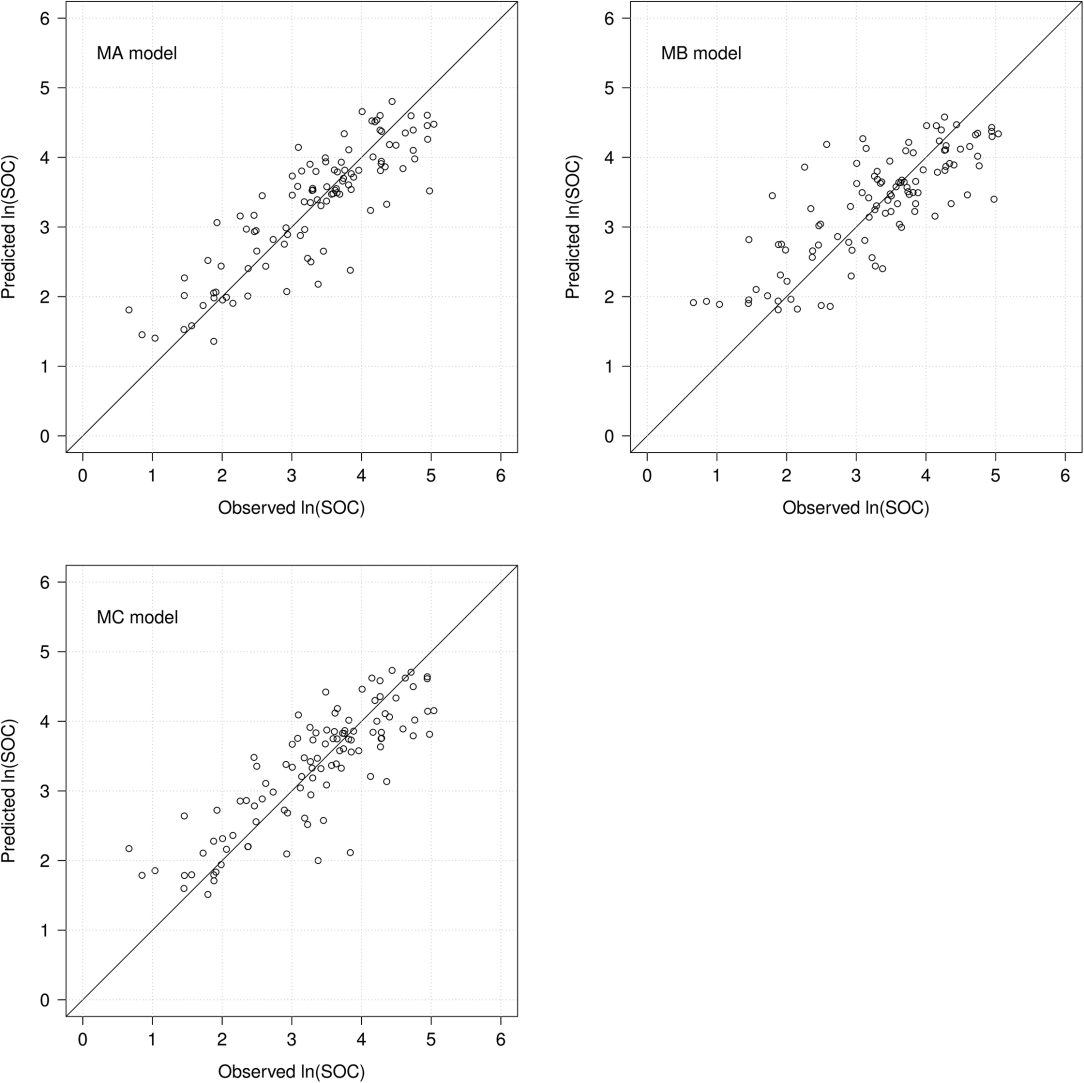
Scatter plots of observed vs. predicted ln(SOC) by three boosted regression tree (BRT) models. MA (topography, climate and Landsat TM imagery); MB (only topography and climate variables); and MC (only Landsat TM imagery).

Fig 2 shows scatter plots of observed and predicted ln(SOC) obtained from three BRT models. These three models underestimated high and overestimated low SOC contents, i.e., the relation has a negative gain, typical result of model smoothing. The MC (imagery-only) model showed the least gain, whereas the MB (topography and climate only model) showed the most. This is consistent with Huang et al. [15] who estimated soil total carbon via 15 m Landsat ETM reflectance data with and without topography variables using multiple regression equations over bare soil. They found that the explained of the variations in total carbon increased from 43% to 60% by combining imagery with topographical data.

**Fig 3:**
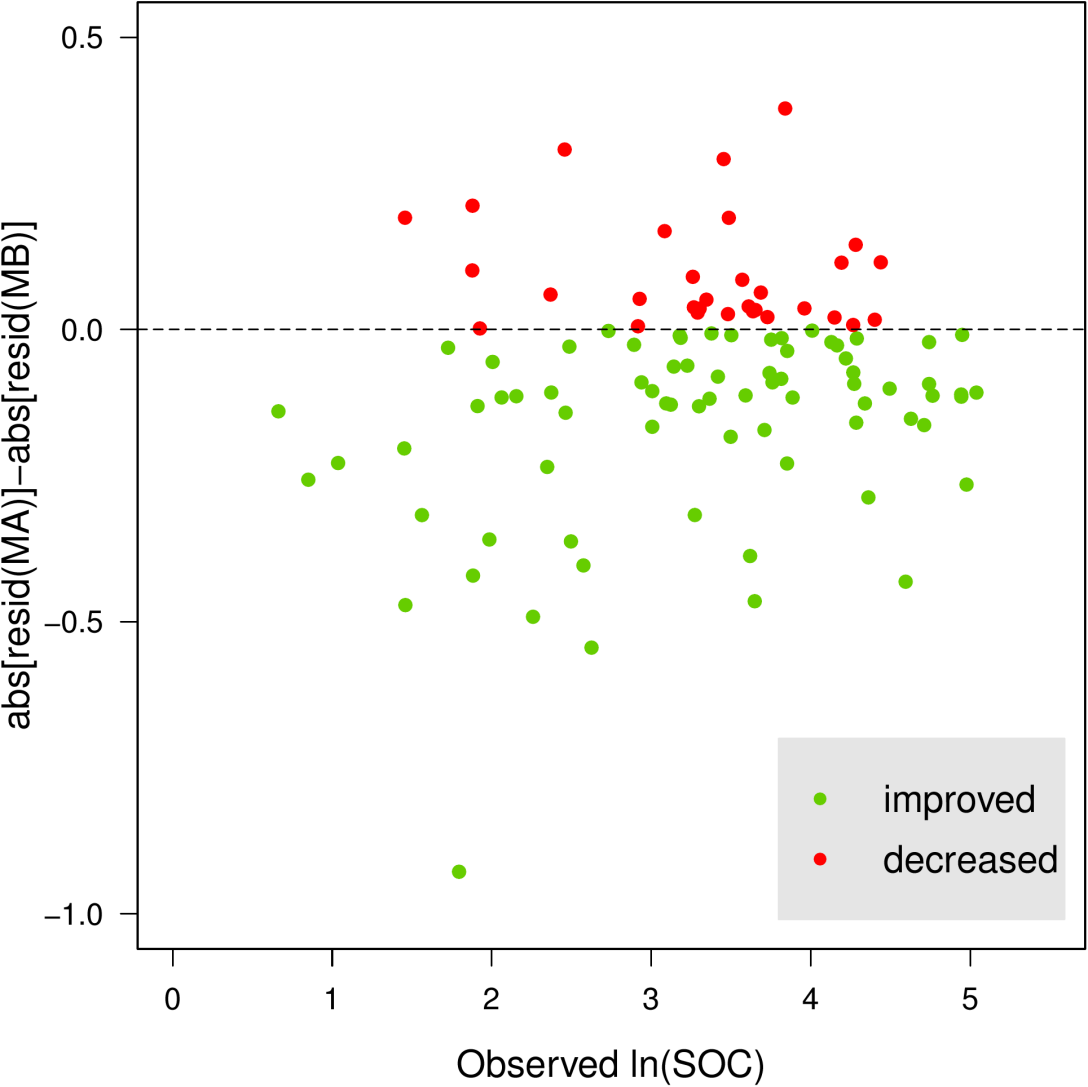
Difference of absolute residual values between MA model (topography, climate and Landsat TM) and MB model (topography and climate). Green: prediction accuracy increased for the site; red: accuracy decreased.

To evaluate the added value of imagery, we calculated the difference values of absolute residuals between MA model and MB model. Fig 3 shows that the cross-validation accuracy of most sites was improved, as evidenced by the smaller residuals.

Our findings demonstrate that multispectral satellite images are practical in predicting topsoil organic carbon with reasonable accuracy in natural vegetation covered regions. The promising predictions might benefit from the strong dependence of SOC on native vegetation intensity and the well correlation between predictors and SOC in this Alpine environment. Our result is supported by the results found by Jaber & AI-Qinna [16] in a semi-arid environment of Jordan, who used six bands (bands 1–5 and 7) of Landsat TM images and stepwise regression to predict SOC content in natural field conditions, obtaining a R^2^ value of 0.22. They attributed the low accuracy to the poor correlation between SOC and reflectance, varying from -0.14 (band 5) to -0.27 (band 3).

Several attempts have been made to predict SOC on bare soils [10, 11, 13, 15]; however these results are not comparable to the present study, since the reflectance and NDVI of this study are largely from vegetated areas, or are showing the contrast between naturally vegetated and bare areas.

**Fig 4:**
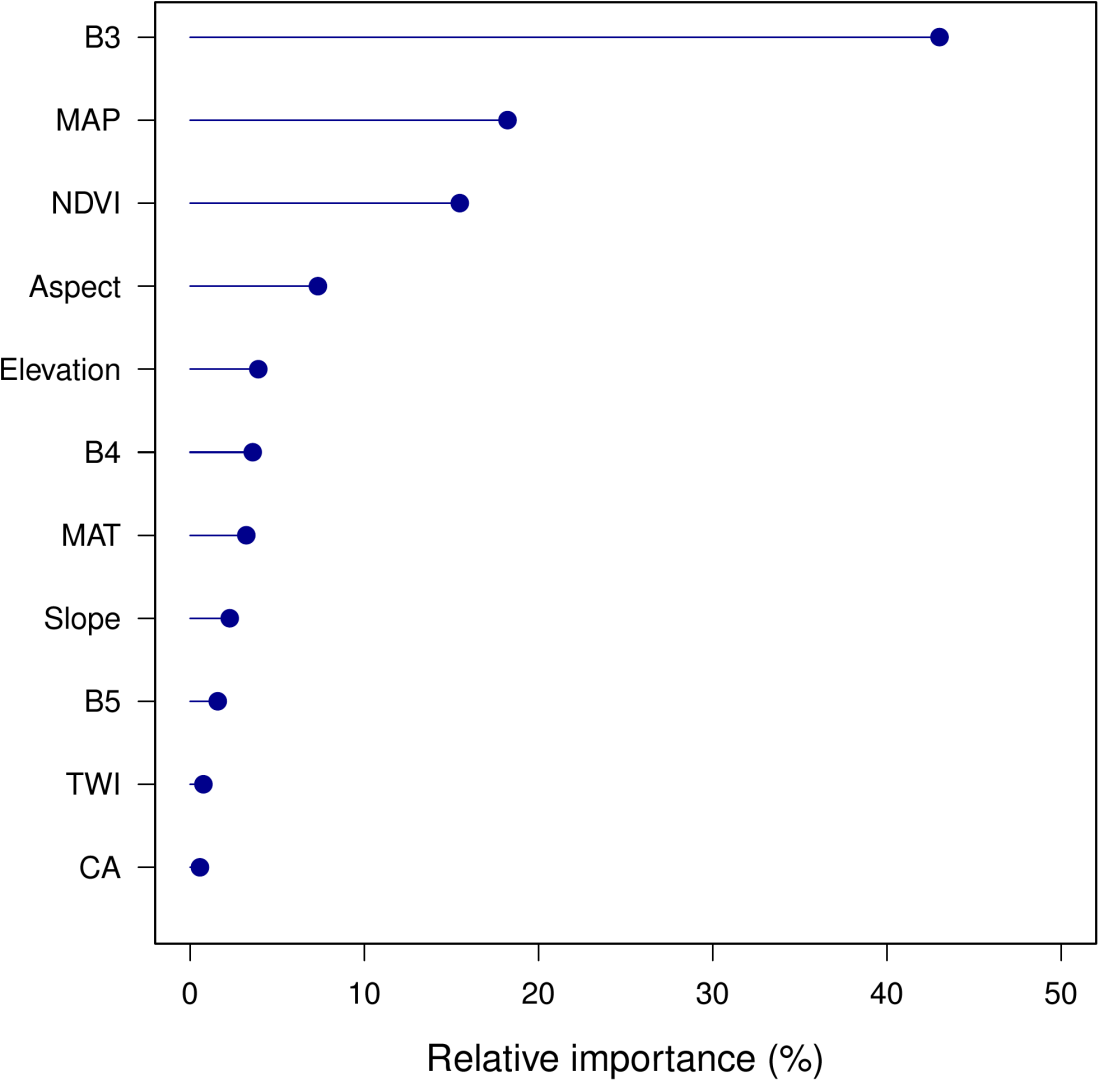
Relative importance of each predictor in the full (MA) model. CA, catchment area; TWI, SAGA wetness index; MAP, mean annual precipitation; MAT, mean annual temperature; B3, Landsat TM band 3; B4, Landsat TM band 4; B5, Landsat TM band 5; NDVI, normalized difference vegetation index.

The BRT model also reports the relative importance of each predictor variable. In the full model, the largest contributions were from B3, MAP, NDVI, aspect and elevation (Fig 4). SOC was mainly explained by Landsat TM imagery (65% relative importance), followed by climate variables (20%) and topography variables (15%). This shows that vegetation, as detected by the imagery, was the most influential factor in predicting SOC content, followed by climate and topography factors. This is expected, since vegetation has been proven to be well-correlated with the spatial patterns of topsoil C, especially in naturally vegetated areas [48]. Remotely-sensed images and derived vegetation indexes have been associated with vegetation cover, vegetation type, biomass and productivity [49–51]. In digital soil mapping procedures, remote sensing images have been used as a proxy for the biosphere as a soil forming factor [5].

**Fig 5:**
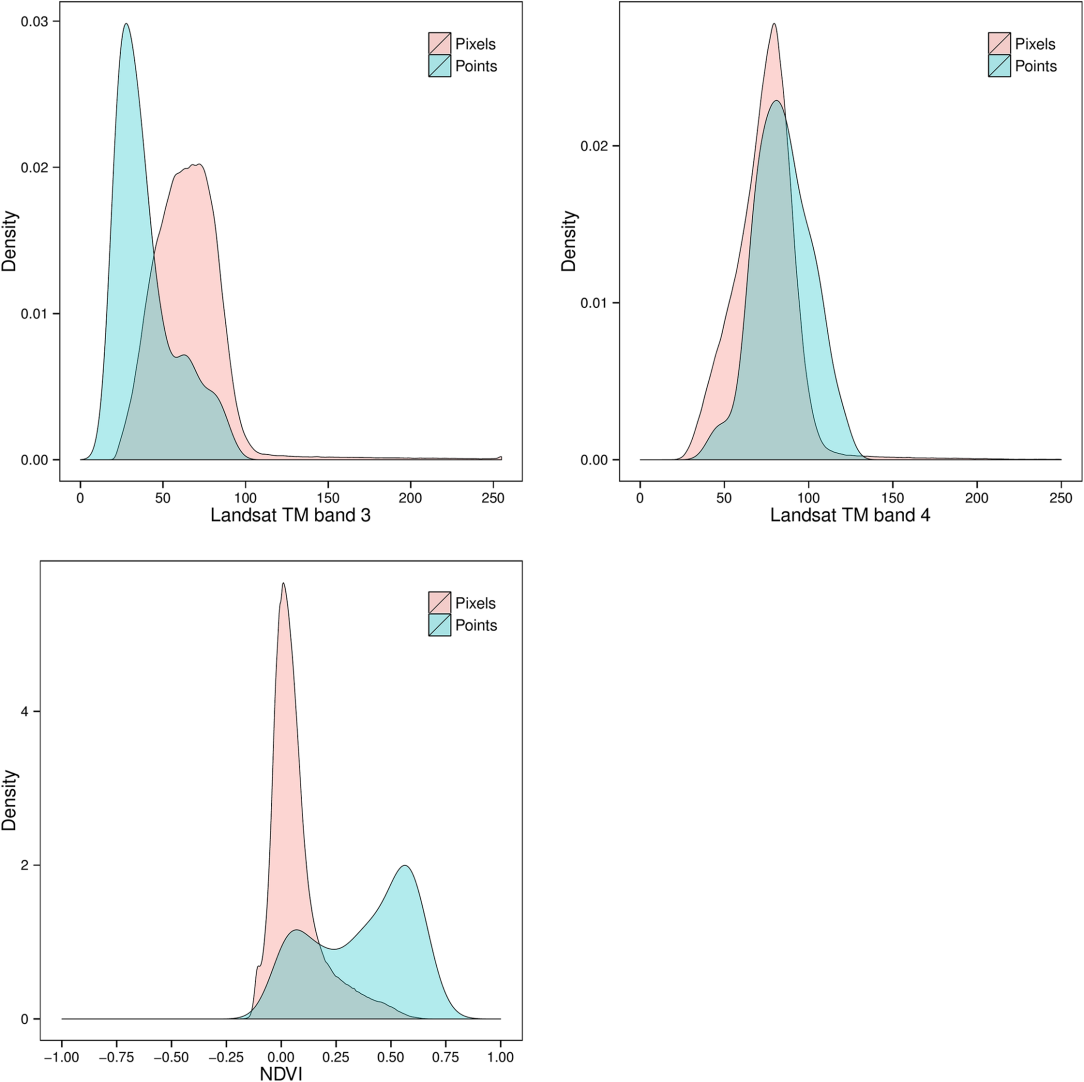
Density plots of Landsat TM band 3, band 4 and NDVI at the pixels. 105 soil samples were taken (pink) and all pixels in the prediction area (blue).

**Fig 6:**
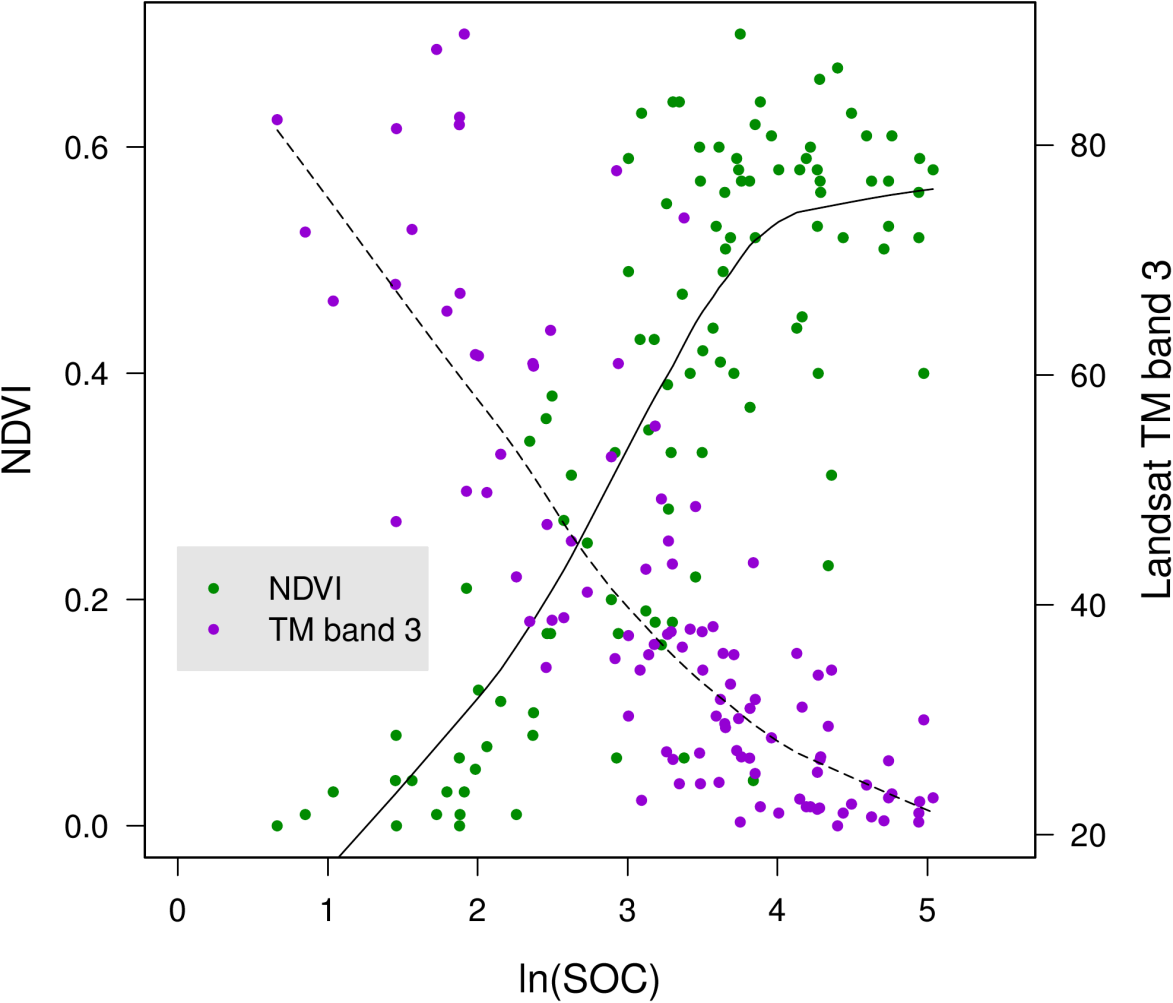
Scatter plot of Landsat TM band 3 and NDVI vs. ln(SOC). It is based on 105 soil samples, with empirical smoothed line.

A surprising result revealed in Fig 4 is that Landsat TM band 3 (red visible) is the most important predictor in the BRT model, much better than NDVI. A single band has no correction for shadow effects nor for non-vegetation (i.e., red colour but not from red phytopigments); indeed this is why ratios such as NDVI were developed. The explanation is shown in Fig 5: the B3 feature-space distribution of the calibration samples is not representative of the full image (prediction area); specifically, there are fewer low values at the profile locations. The points of B4 are slight biased towards higher values than pixels. Thus, NDVI biases in the higher values. Partly this is because no dark-colored bare rock areas (low reflectance) were sampled for SOC; further, no soils were sampled in areas covered by water. On the other hand, there is a saturation effect in detecting SOC from NDVI and B3 at highly-vegetated areas (Fig 6). Even though the Pearson correlation is almost as high for NDVI and SOC (r = 0.79) as for B3 and SOC (r = -0.82), NDVI shows a lower sensitivity than B3 when they are applied to predict high SOC content. Therefore, B3 is preferred to NDVI in the BRT model.

**Fig 7:**
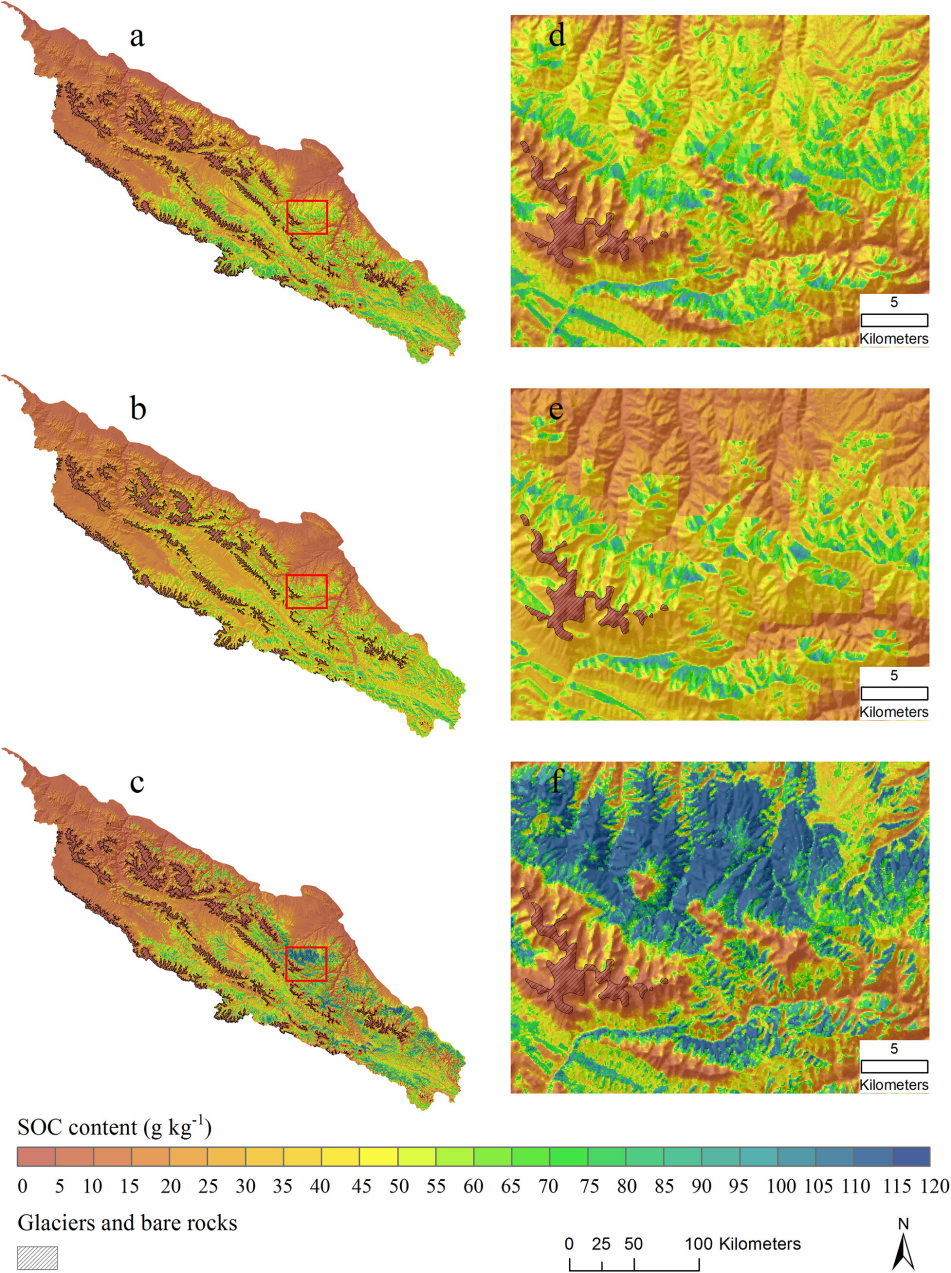
Distribution maps of topsoil organic carbon (g kg^-1^) derived from three boosted regression tree (BRT) models with a masking layer of glaciers and bare rocks (overlaid hillshading). a) MA model included all predictors (topography, climate and Landsat TM imagery); b) MB model included only topography and climate variables; and c) MC model included only Landsat TM imagery (B3, B4, B5 and NDVI); d), e) and f) small areas outlined with red color in left large areas for showing detail information.

**Fig 8:**
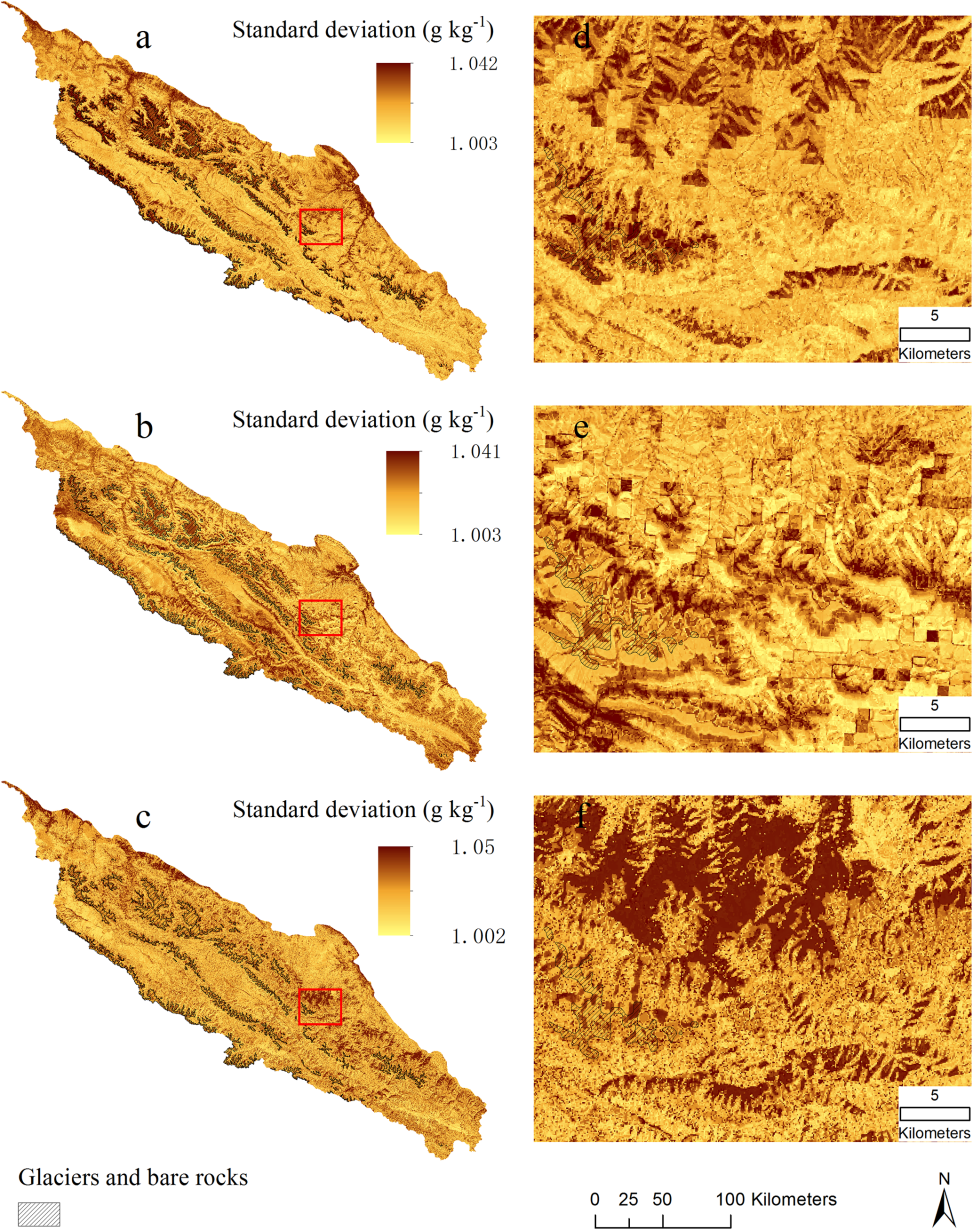
Standard deviation maps of predicted topsoil organic carbon (g kg^-1^). a) MA model included all predictors (topography, climate and Landsat TM imagery); b) MB model included only topography and climate variables; and c) MC model included only Landsat TM imagery (B3, B4, B5 and NDVI); d), e) and f) small areas outlined with red color in left large areas for showing detail information.

The predicted distributions of SOC content and standard deviation from three BRT models are shown in Fig 7 and Fig 8. Areas of glaciers and bare rocks are figured out from Landsat TM imagery using a supervised classification method and masked out in Fig 7 and assigned zero SOC values. Spatial patterns of SOC are obviously closely related to vegetation (compare Fig 1). High SOC contents are found in the south-eastern mountains, which have the densest vegetation cover, according to Jin et al. [52] who quantified vegetation distribution in the Qilian Mountains and found the densest vegetation cover between 3200 and 3600 m elevation. Low SOC contents were in the northern and north-western parts, which are dominated by low productivity plants such as desert-grassland and dry shrub-grassland [52].

The mean values and SD values of predicted SOC content were 26.78 and 20.81 g kg^-1^ for MA model, 26.08 and 14.82 g kg^-1^ for MB model and 28.27 and 27.12 g kg^-1^ for MC model, respectively. Notable is that the imagery-only model (MC) has a somewhat higher mean and a much larger SD than the other models; that is, its spatial pattern is more variable. Fig 7D–7F shows a large area where MC model predicts up to 60 g kg^-1^ higher than MA model. In the MC model (variables of Landsat TM), the SOC prediction completely depends on the reflectance values of pixels. The sites have low reflectance values of B3 and high values of B4 and NDVI, are tend to be estimated with high SOC content in this Model. However, optical remote sensing is found to be sensitive to near-surface moisture and mountain shadows that could lead to biased reflectance and thus bring uncertainties in SOC prediction. In MA model, the effect of Landsat imagery on SOC is mediated by adding topography and climate variables. As such, these added variables can diminish the uncertainties of remote sensing imagery in full model and improve prediction accuracy as shown in Table 3.

**Fig 9:**
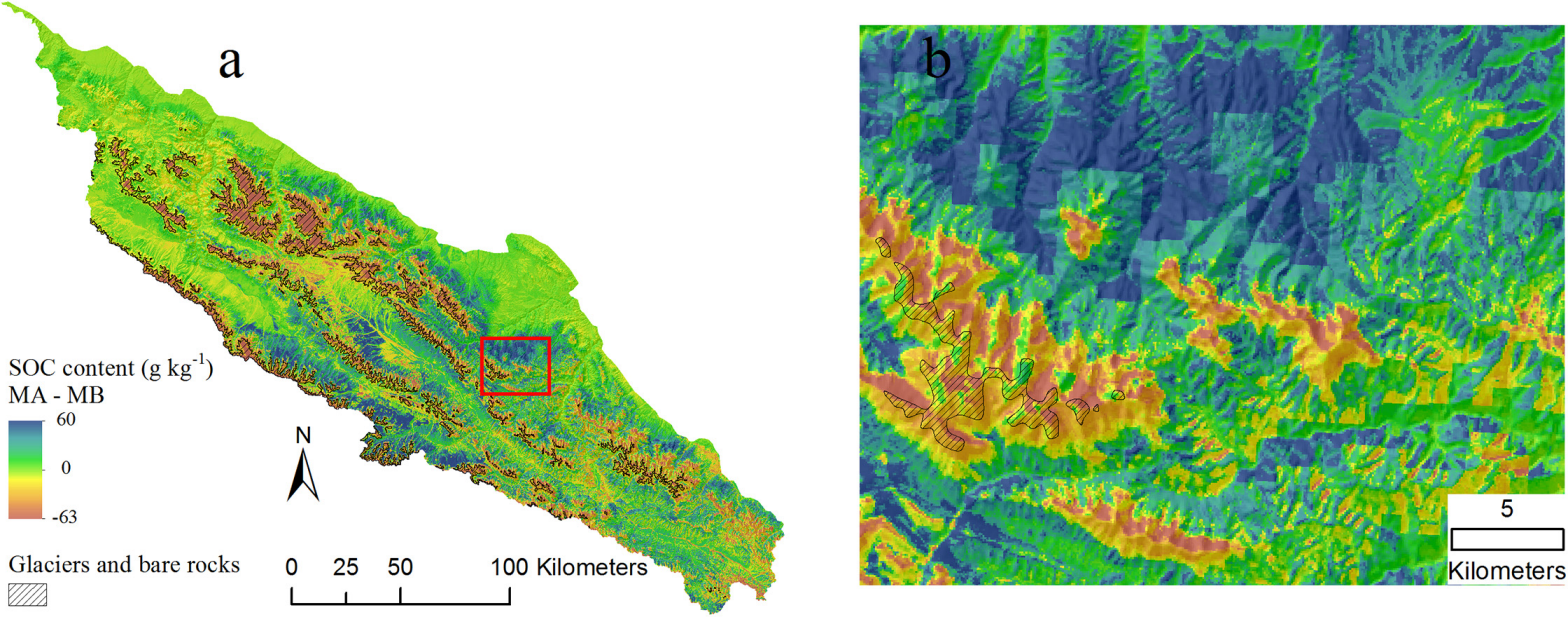
Difference map of soil organic carbon (g kg^-1^) derived from MA and MB models (overlaid hillshading). MA model included all predictors (topography, climate and Landsat TM imagery); MB model included only topography and climate variables.

Fig 9 shows the difference in predicted SOC content based on the MA (full) and MB (topography and climate only) models. It is clear that adding multispectral Landsat TM imagery (model MA) provides more detail especially in the high SOC areas of model MB. By adding Landsat TM imagery, SOC in areas covered by glaciers and bare rocks dramatically decreases, with a corresponding increase in areas with high vegetation cover. The maps from the MA (full) and MC (imagery only) models are similar. Though SOC is well-correlated with precipitation and air temperature (Table 2), these climate features operate over wide areas and thus are too coarse to explain local SOC variability. This is where fine resolution remote sensing data can improve prediction (as shown in the visualization) due to its high resolution and relation to vegetation cover.

**Fig 10:**
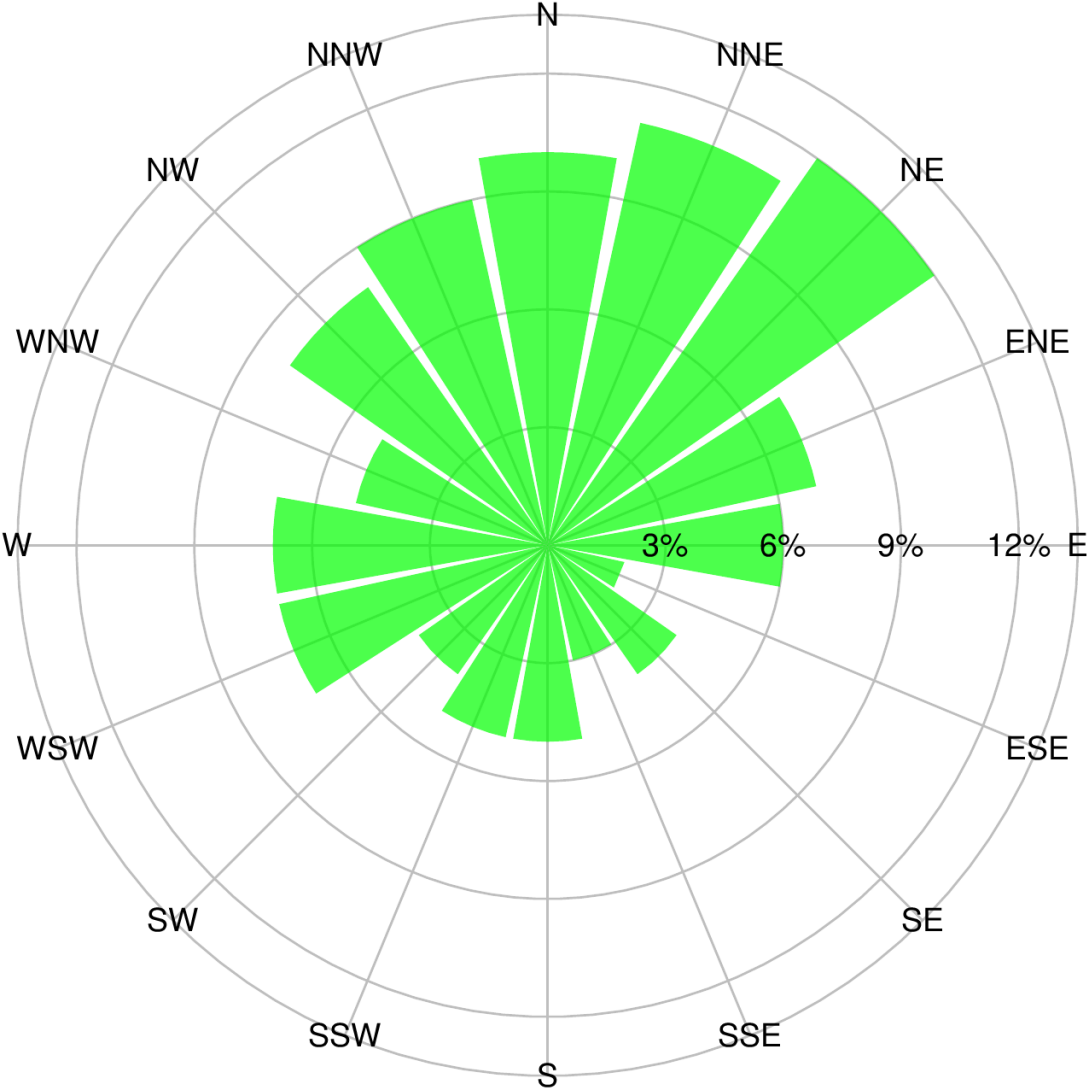
Rose diagram of the aspect of 105 sample sites. The proportion of samples facing specific aspect was shown as the length of green bar.

Despite the success of Landsat TM imagery in this study, it is important to note that using only imagery for prediction has some drawbacks. In high-relief areas reflectance is influenced by shadow caused by high relief and clouds [53], leading to confusion for image classification and land cover recognition [53–55]. In our study, SOC on north-facing slopes are predicted to be somewhat higher than on the south-facing slopes (Fig 7); this is consistent with field observations and the low but significant correlation between SOC and aspect (r = -0.22, Table 2). However, the SOC content distribution map from the MC (imagery only) model seems to be influenced by mountain shadows, so that very high SOC contents are predicted in the N aspect positions (Fig 7). Note that the Landsat 5 overfly time is nominally 0945, i.e., mid-morning. The mosaic is from July to September, i.e., mid to late summer; in mid-August the Sun at 0945 has azimuth of 100° (i.e., ESE) and elevation of 36° (http://www.esrl.noaa.gov/gmd/grad/solcalc/azel.html), meaning that steep slopes facing WNW will be in shadow and have low reflectance, thus exaggerating the actual effect of aspect. NDVI is expected to correct for shadow effects, since it is normalized by the same bands as used in the difference. However, the samples are not evenly distributed as shown in the rose diagram of the aspect of the sample sites (Fig 10). They are mostly NNW to NE facing, and there are few samples facing the sun at the time of acquisition. Thus, the shadow correction is not so important in this study. Highly variable topographical attributes of plateau terrain cause difficulties in mapping SOC based only on remote sensing imagery. Topography is proved to be a valuable predictor for improving prediction accuracy from remote sensing data and resulting in more reliable predictions in such areas. Thus, topographical attributes are recommended in addition to remote sensing data for accurate SOC mapping in Alpine environments.

## Conclusions

This study shows that low-cost, easily-obtainable multispectral optical and near IR imagery such as Landsat TM can by itself provide a spatially-detailed and reasonably accurate map of topsoil SOC in high-relief, naturally-vegetated Alpine areas. Adding standard topographic and climatic covariates somewhat improves the model, but not dramatically. The improvement in detail is probably due to some compensation for shadow effects on images. We conclude that multispectral imagery should be used for digital soil mapping of topsoil SOC in Alpine environments.

## Supporting Information

S1 Dataset Soil organic carbon (SOC) and coordinates on soil sample sites. (XLS)
